# Patient experience of the informed consent process during acute myocardial infarction: a sub-study of the VALIDATE-SWEDEHEART trial

**DOI:** 10.1186/s13063-020-4147-0

**Published:** 2020-03-06

**Authors:** Anneli Olsson, Camilla Ring, Johan Josefsson, Annika Eriksson, Rebecca Rylance, Ole Fröbert, Stefan James, David Sparv, David Erlinge

**Affiliations:** 1 Department of Cardiology, Clinical Sciences, Lund University, Skane University Hospital, SE-221 85 Lund, Sweden; 2 Department of Cardiology, Norrland University Hospital, Umeå, Sweden; 3 Department of Coronary Heart Disease, Örebro University Hospital, Örebro, Sweden; 4 Department of Medical Sciences, Cardiology, Uppsala University, Uppsala, Sweden

**Keywords:** Myocardial infarction, Informed consent

## Abstract

**Objective:**

We aimed to assess the patient experience of informed consent (IC) during acute myocardial infarction (AMI) in a sub-study of the VALIDATE-SWEDEHEART trial. The original trial compared two anticoagulant agents in patients undergoing coronary intervention. A witnessed oral IC was required prior to randomization in patients with ST-segment elevation myocardial infarction, which was subsequently complemented with a written IC after percutaneous coronary intervention. Written consent was obtained before angiography in patients with non-ST-segment elevation myocardial infarction.

**Background:**

The IC process in patients with AMI is under debate. Earlier trials in this population have required prospective consent before randomization. A trial published some years ago used deferred consent, but the patient experience of this process is poorly studied.

**Methods:**

A total of 414 patients who participated in the main trial were enrolled and asked the following questions: (1) Do you remember being asked to participate in a study? (2) How was your experience of being asked to participate; do you remember it being positive or negative? (3) Would you have liked more information about the study? (4) Do you think it would have been better if you were included in the study without being informed until a later time?

**Results:**

Of these patients, 94% remembered being included; 85% of them experienced this positively, 12% were neutral and 3% negative. Regarding more information, 88% did not want further information, and 68% expressed that they wanted to be consulted before inclusion. Of the patients, 5% thought it would have been better to have study inclusion without consent, and 27% considered it of no importance.

**Conclusion:**

It is reasonable to ask patients for verbal IC in the acute phase of AMI. Most patients felt positively about being asked to participate and had knowledge of being enrolled in a scientific study. In addition they objected to providing IC after randomization and treatment.

**Trial registration:**

VALIDATE-SWEDEHEART European Union Clinical Trials Register: 2012-005260-10. ClinicalTrials.gov: NCT02311231. Registered on 8 Dec 2014.

## Introduction

Clinical research is essential for the continuous development of modern medicine, but it poses challenges regarding the mandatory informed consent (IC) process for patients in acute and critical conditions [1].

Even though legislation and ethical guidelines [2] offer guidance, conducting clinical trials in patients with certain medical conditions is a difficult balance of timely treatment and written IC [3, 4]. According to the Declaration of Helsinki [2], patients who are capable of providing IC shall do so. If that is not a possibility, the principal investigator must seek consent from a legally authorized representative. Exceptions in informed consent (EFIC) may be justified when no legally authorized representative is available and if the inclusion process cannot be delayed, i.e. in an emergency situation. Several criteria must be fulfilled, such as minimal risk for the patient, an intention to promote health for the group and that equivalent research could not be performed with only capable persons [2].

In patients with ST-segment elevation myocardial infarction (STEMI), recent publications suggest that EFIC as well as standard IC may be insufficient [3, 4]. Patients with STEMI in general are mentally and physically stressed, often in pain and in addition treated with analgesics and sedatives [5]. Also, in order to swiftly perform myocardial salvage by primary percutaneous coronary intervention (PPCI), time is limited for the research IC process.

The management of the IC process in patients with acute myocardial infarction (AMI) has been debated for several decades [6, 7]. In a Swedish study investigating the opinion of more than 700 cardiologists, 44% did not consider patients with an AMI to be in a position to be asked about trial participation in the acute phase [8].

Knowledge of AMI patient perspectives regarding the IC process for research is limited and inconclusive. Previous studies indicate that patients have difficulties understanding study details and impaired cognitive capability in the acute phase [4, 9, 10]. Furthermore, few patients read the written information before consenting and prefer verbal information [10, 11]. Nevertheless, the majority of patients consider themselves well suited to take part in an enrolment decision [4, 11].

The current common practice in AMI trials is to obtain prospective, most often written IC [12], but this has recently been debated [3, 12–15]. In the HEAT-PPCI trial, comparing bivalirudin and heparin, the ethics committee in the UK approved a delayed IC process [16]. In accordance with the study protocol, no attempt was made to discuss the trial with the patient or to seek consent prior to randomization. When asked to provide IC to remain in the study the day after randomization, only 4 out of 1829 patients refused consent.

In our registry-based randomized clinical trial (RRCT) VALIDATE-SWEDEHEART [17] (the Bivalirudin versus Heparin in ST-Segment and Non–ST-Segment Elevation Myocardial Infarction in Patients on Modern Antiplatelet Therapy in the Swedish Web System for Enhancement and Development of Evidence-based Care in Heart Disease Evaluated according to Recommended Therapies Registry Trial), we compared the same pharmaceutical agents as in the HEAT-PPCI trial. In patients with STEMI, a witnessed oral IC was required prior to randomization, and this was subsequently complemented with a written IC after the percutaneous coronary intervention (PCI). A number of endpoints were only [18] followed up in the SWEDEHEART registry, but study-specific research nurses also screened for clinical endpoint events by contacting patients by telephone 7 and 180 days after PCI [19].

The aim of this pre-specified sub-study was to study patient experience in relation to the IC process in the VALIDATE-SWEDEHEART trial.

## Methods

### Design

The VALIDATE-SWEDEHEART trial [17] was a registry-based, multicentre, randomized, controlled open-label clinical trial. The trial was approved by the ethics committee of Lund University, Sweden and by the Swedish Medical Products Agency. With assistance from all investigators, an executive committee was responsible for the design, conduct and reporting of the study.

### Patients

Patients admitted to the hospital with a diagnosis of STEMI or non-ST-segment elevation myocardial infarction (NSTEMI) for whom urgent PCI was planned were eligible for inclusion in the trial according to previously published inclusion and exclusion criteria [19]. IC was obtained in the coronary catheterization laboratory. In the STEMI group, patients were asked for witnessed oral consent prior to PCI. Following the procedure, this was complemented by a written confirmation. In the NSTEMI group, a written IC was mandatory before randomization.

### Procedure

This study was performed at three of the hospitals participating in the main trial. During the telephone follow-up call 1 week after inclusion according to the main study protocol, patients who had already consented to participate in the main trial were consecutively enrolled in this sub-study. They were asked to participate in a survey comprising four questions: (1) Do you remember being asked to participate in a study? (2) How was your experience of being asked to participate; do you remember it being positive or negative? (3) Would you have liked more information about the study? (4) Do you think it would have been better if you were included in the study without being informed until a later time? These four questions were decided mainly through empirical findings from previously performed trials in this population, patient input and the purpose of this trial.

### Data collection

The telephone follow-up call was conducted by study-specific research nurses. If patients could not be contacted, a letter was sent with a request to contact the research nurse. The collection of data was performed using a pre-defined matrix, where the answers were compiled and processed subsequently.

### Statistical analysis

Categorical variables were analysed with the chi-square test, evaluating differences in proportions between groups. If the number of observations was too few, Fisher’s exact test was used. A *p* value of ≤0.05 was considered statistically significant. Exploratory pre-defined sub-group analyses regarding STEMI/NSTEMI diagnosis, gender and age were performed using the same statistical methods.

## Results

### Patient characteristics

Between February 2015 and January 2016, a total of 414 patients (6.9% of the main trial population) who had already consented to participate in the main trial were included in the present sub-study (see Table 1). None of the 414 patients who were contacted in this sub-study declined to answer the study questions. Two patients had hearing impairment and were excluded from the final analysis due to inconclusive telephone communication. The baseline characteristics in this sub-study were similar to those of the main trial with regard to age, sex and comorbidities [17]. Of the patients included in the sub-study, 56.5% were diagnosed with STEMI compared to 50% in the main trial (Table 1).

**Table 1:** Baseline characteristics

Characteristics	Total 414
STEMI, number (%)	234 (56.5)
Male sex, number (%)	309 (74.6)
Age	
Median, years	67
Interquartile range (IQR), years	59–73
Body mass index	
Median	26.4
IQR	24.2–29.4
Weight < 60, number (%)	20 (4.8)
Previous smoker, number (%)	154 (37.2)
Current smoker, number (%)	95 (23.0)
Diabetes, number (%)	44 (10.6)
Hypertension, number (%)	209 (50.5)
Hyperlipidemia, number (%)	108 (26.1)
Previous myocardial infarction, number (%)	61 (14.7)
Previous percutaneous coronary intervention, number (%)	54 (13.0)
Previous coronary artery bypass grafting, number (%)	19 (4.6)
Cardiopulmonary resuscitation before arrival at the catheterization laboratory, number (%)	6 (1.5)
Killip class II, III or IV, number (%)	13 (3.1)

In the main trial, 93.9% of the patients asked for study participation accepted enrolment. Of the patients who consented to the main trial, 86.2% could be reached for the follow-up call after 7 days.

### Informed consent

**Fig. 1:**
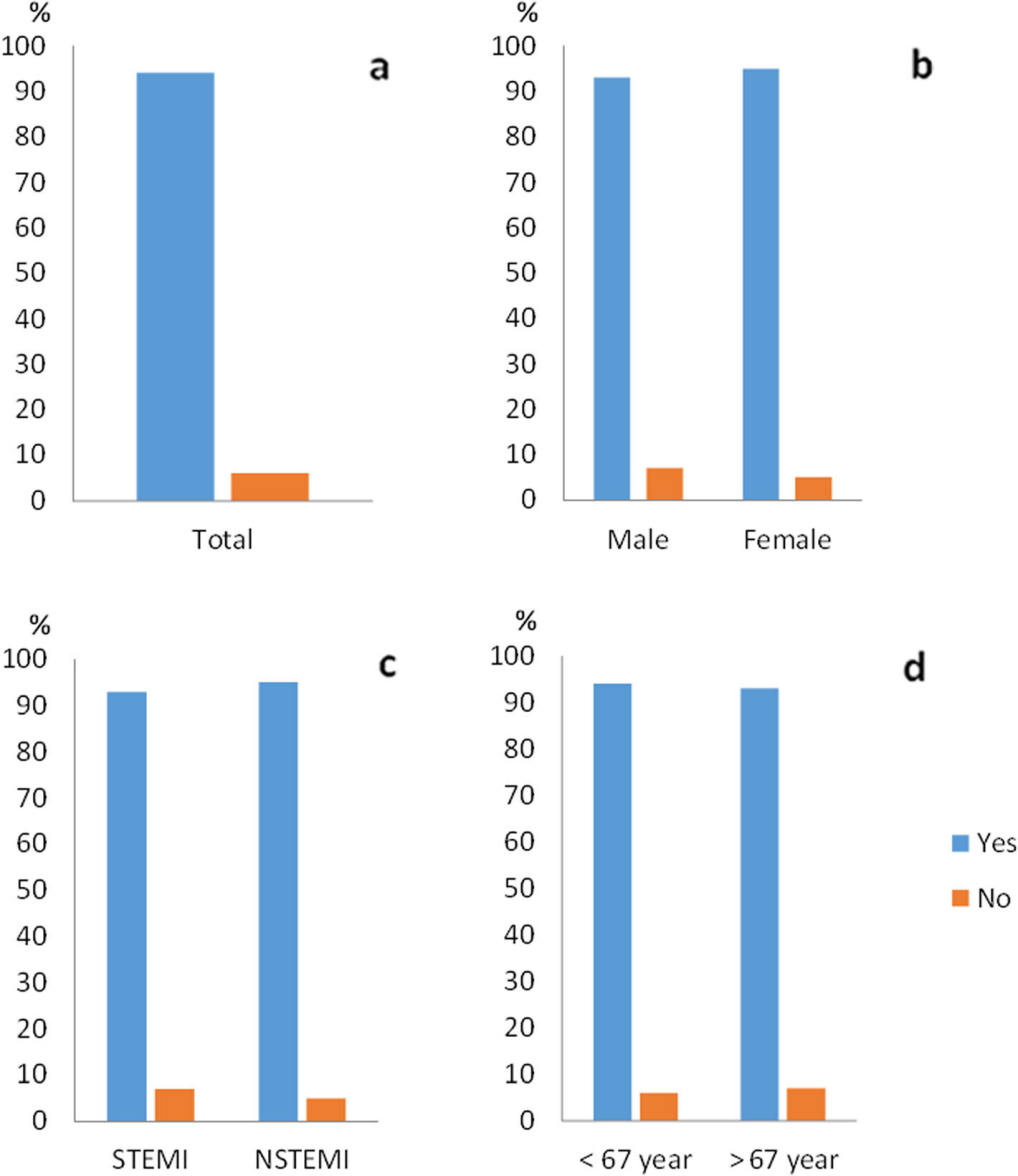
Remembrance of the informed consent process in the main trial population (**a**) and in the sub-groups of gender (**b**), clinical presentation (**c**) and age (**d**). There were no significant differences in any of the sub-groups

**Fig. 2:**
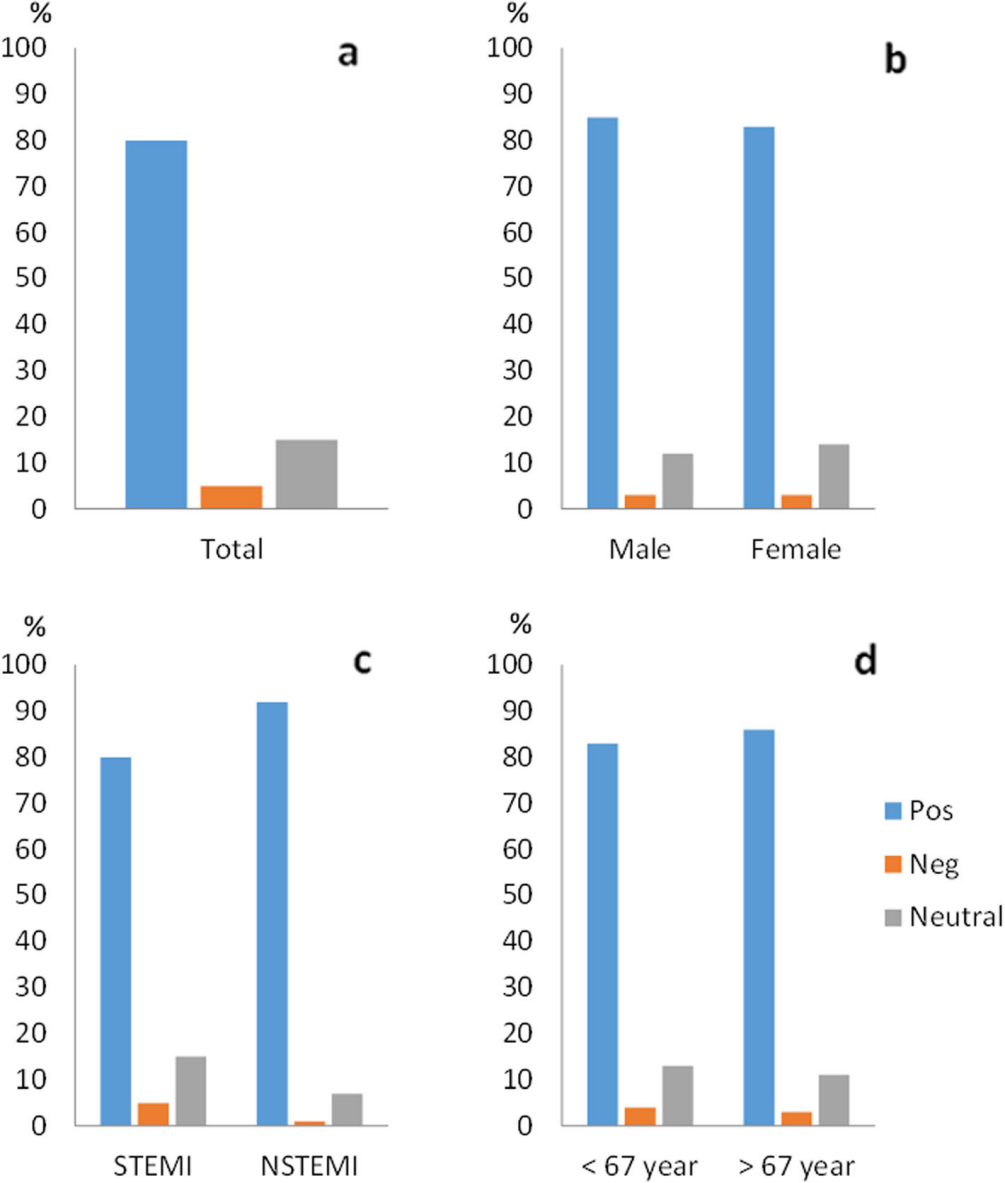
Patients’ experience of being asked in the main trial population (**a**) and in the sub-groups of gender (**b**), clinical presentation (**c**) and age (**d**). The STEMI group were significantly less positive than the NSTEMI group, *p* = 0.005

**Fig. 3:**
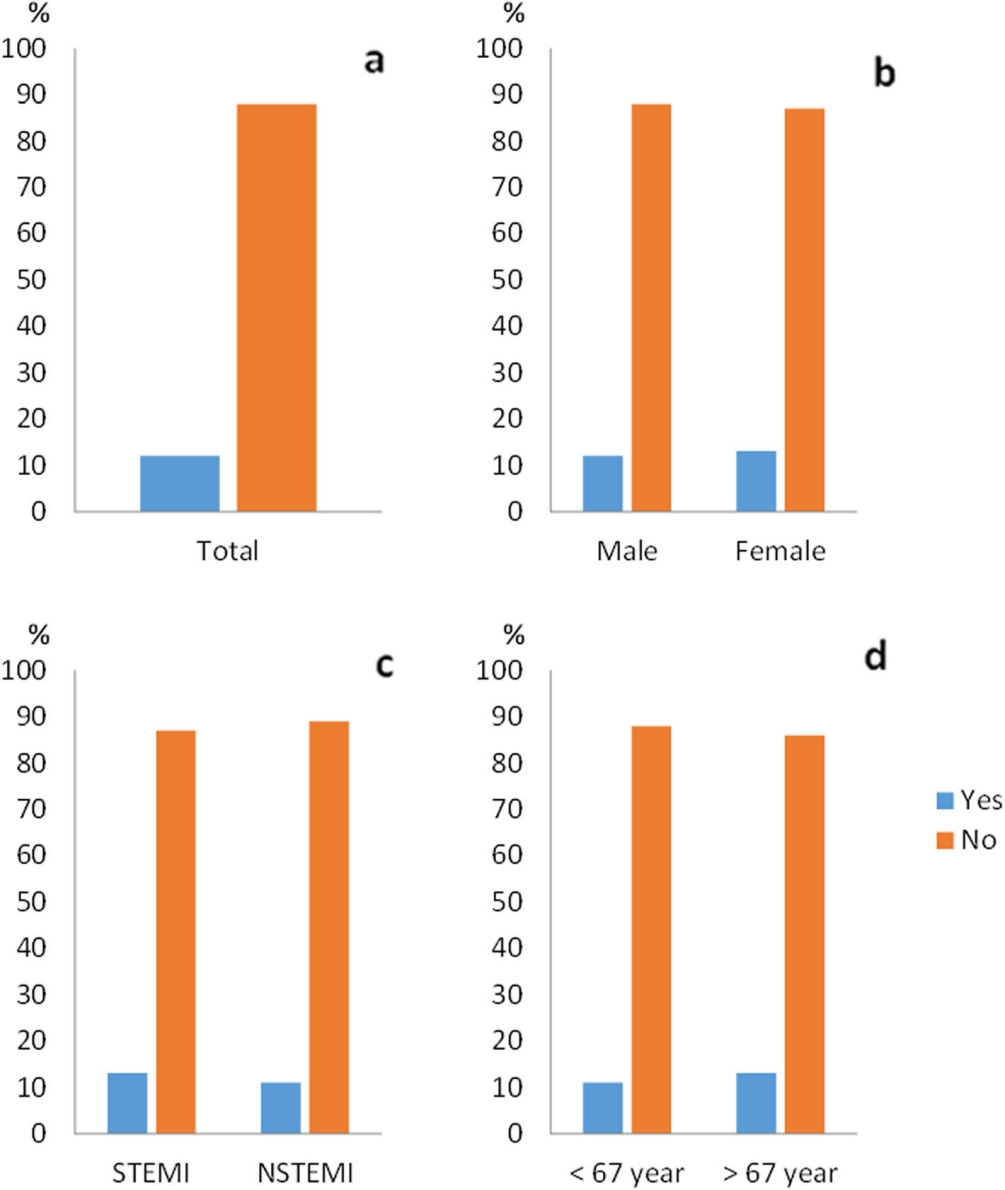
**a**–**d** as in Fig. 1. Patients’ preference to have more information. There were no significant differences in any of the sub-groups

**Fig. 4:**
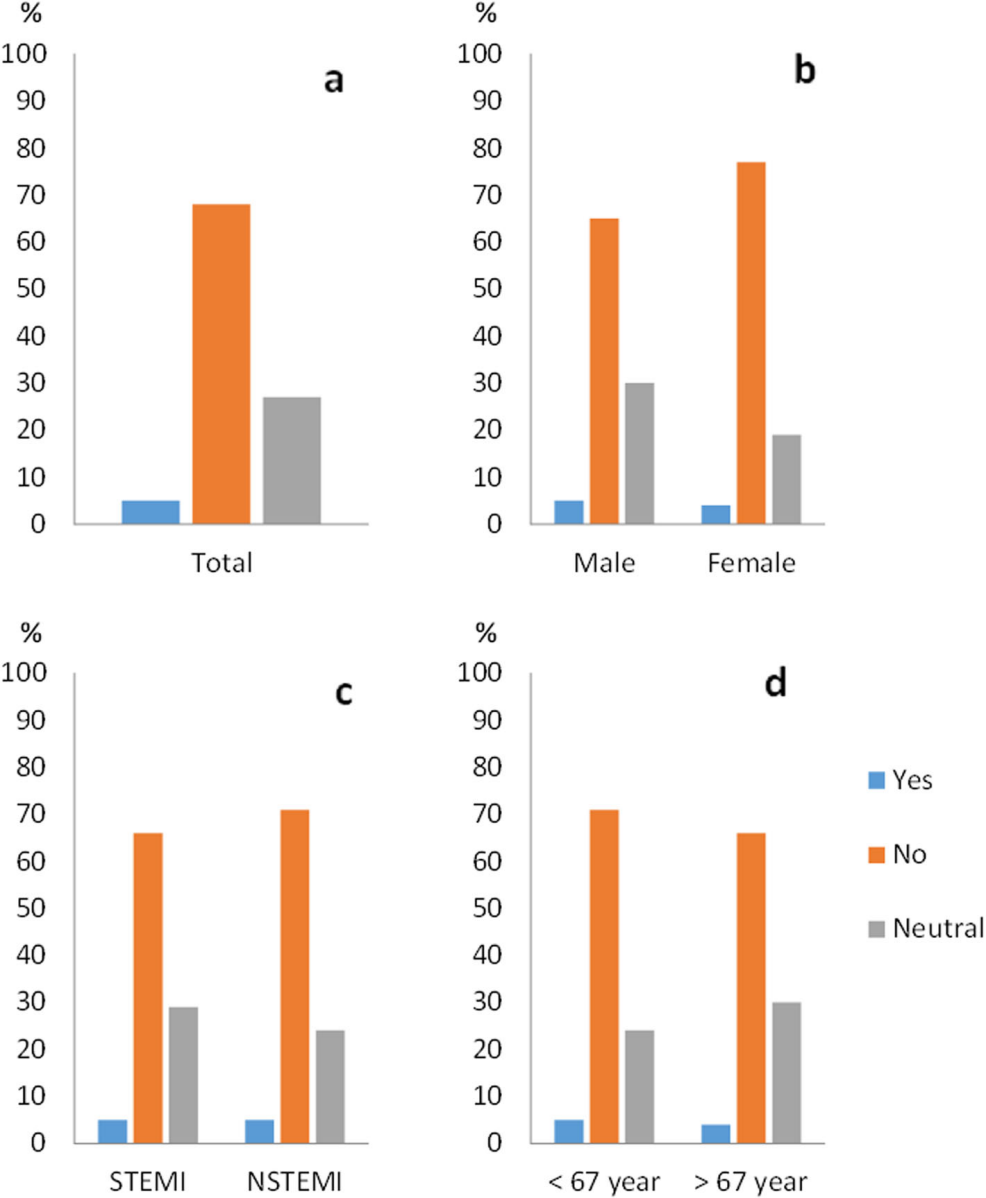
**a**–**d** as in Fig. 1. Patients’ view of delayed consent. **b** Females tended to be more negative to delayed consent than men, *p* = 0.06. **c** There were no significant differences between STEMI and NSTEMI or in the age sub-group

Among all patients in this sub-study, 388 (94%) recalled being included in a research study. Of these, 85% expressed a positive opinion about being asked to participate in a study, 12% were neutral and 3% described this as a negative experience. Of the patients, 88% did not wish to have received more comprehensive study information during the initial oral IC. Sixty-eight percent wanted to be consulted before study inclusion, 5% preferred post hoc consent while 27% were neutral (Figs. 1, 2, 3 and 4).

### Sub-groups

There were no significant differences in the sub-groups regarding memory of study inclusion. The STEMI group stated a significantly less positive experience of the IC process compared to the NSTEMI group (79% versus 91%, *p* = 0.005) (Fig. 2c). No sub-group differences in the need of information appeared. For the question regarding deferred consent, women were more often negative to deferred consent compared to men, but this was not statistically significant (77% versus 65%, *p* = 0.059) (Fig. 4b). We did not see any differences in relation to age.

## Discussion

In this clinical study evaluating patient experience of the IC process in the VALIDATE-SWEDEHEART trial, the majority of patients remembered being asked to participate in the study. Also, more than 80% felt positively about being asked for IC in the acute situation; only 5% would prefer to be included and receive information afterwards. Most patients considered information about the study provided in the acute phase to be sufficient.

### Memory

The process of information retrieval in the acute setting of an AMI has previously been studied with substantial variability. In an interview study (*n* = 20) by Dickert and colleagues [4], merely 55% of the patients could recall being asked to participate in a study. In the larger HERO-2 sub-study [20] (*n* = 399), the results concerning remembrance were similar to our findings: 94% of the enrolled participants remembered that information about participation was given during the acute phase of AMI. It is reasonable to assume that in a larger clinical population, most patients will remember the consent process regardless of stress, anxiety and opioid medications. Another important issue is what patients actually remember about the IC process. This study was not designed to investigate this, but previous findings indicate that remembrance in general is fragmentary and focused on several different aspects [4, 10].

### Experience of being asked

In this study, the vast majority of patients did not experience the IC process in a negative way. According to previous findings, the main reasons for participating in a trial are a notion about being able to help and to serve with a purpose of improvement of healthcare. The sense of positivity and understanding may differ depending on the protocol and complexity of the trial. In a questionnaire based on a survey by Gammelgaard et al. [11], investigating the IC process in DANAMI-2 [21], 26% of patients found it unacceptable to make a decision about study participation in the acute phase of an AMI. The discrepancy between our study and the DANAMI-2 cohort could be attributed to the difference in investigating two well-known and commonly used pharmaceuticals (heparin and bivalirudin) compared to investigating standard treatment versus a new technique (fibrinolysis compared to PCI). Also, the configuration of the questions could affect the different results.

A small number of responders expressed a negative feeling about having been asked to participate in the study. Previous findings have described limited time for the IC process as a negative predictor of experience. Williams et al. [20] found that 12% of the patients considered the timing of enrolment inappropriate. In the original trial, 6.1% declined participation. We did not study the reason for this, but findings from the HERO-2 consent sub-study [20] reported that 61% of the patients who declined participation thought that the time allowed for considering consent was too short. In the same study, the corresponding number among patients who gave consent was 25%. This indicates that patients who decline consent feel stressed and, in concordance with previous findings, consider reading and signing a form a complicated and unwanted task [10]. This makes the overall positive results in our sub-study explicable, since the patients with STEMI could read and sign in a calm environment after the acute phase.

### Information

Eighty-eight percent of the patients considered the short verbal information adequate. It seems that the abbreviated IC process is satisfactory if complemented with written information afterwards. Previous studies support this conclusion, since few patients find it possible to read and comprehend written information during an acute phase [11, 20].

Other studies [14, 22] have demonstrated that patients’ confidence in healthcare professionals is generally strong, and this might be an explanation for the patients’ satisfaction and trust in verbal information in the acute phase of an AMI.

### Deferred consent

Prompted by the debate of deferred IC, we also asked participants if they preferred being asked after randomization and PCI. A majority of the patients were negative to post hoc consent and wanted to be involved early in the decision phase, as has been demonstrated in a previous study [4]. Thus, our findings do not support the use of deferred consent in AMI trials without patient involvement pre-randomization.

### Ethical considerations

The informed consent (IC) process is an ethical and legal requirement for research in humans, and bypassing prospective consent brings up ethical challenges. Exceptions for requiring IC or deferred consent are primarily used in trials including patients with cardiac arrest, haemorrhagic shock or traumatic brain injury [23]. European Union (EU) regulations [24] offer advice for ethics committees regarding clinical trials in emergency situations (article 35). In Sweden, this EU article has not yet been implemented in legislation, so far research including persons who are incapable of giving IC in Sweden is not permitted.

Global discussions have been held concerning whether patients with AMI are capable of making informed decisions or not. The population is not a homogenous group. Impaired cognitive capability is present among several patients in the acute phase; nevertheless, the majority of patients consider that they are well suited to take part in an enrolment decision.

In the UK, where the HEAT-PPCI trial was performed, the argument for deferred consent was mainly the life-threatening situation in combination with the minimal risk of comparing two well-established drugs [16]. The advantage of using deferred consent is a more comprehensive recruitment of an unselected study population, providing data which can be more easily generalized to everyday patient care [25]. The potential risk is to include an unwilling patient. In this sub-study including patients with similar conditions as in HEAT-PPCI, we conclude that patients want to be involved in the IC process in early stages. A dialogue between the physician and the patient is essential. Despite the emergency setting, patients can receive brief trial information and an opportunity to decline participation. Fully written IC is not feasible for many reasons in the emergency setting. This sub-study evaluates the verbal consent, which many patients found to be sufficient.

### Limitations of the study

The present study has several limitations. The structured telephone survey including four questions did not allow further exploration of participants’ attitudes towards the use of deferred consent. The choice of wording of the questions may have affected the results. We did not use independent interviewers, and study-specific research nurses involved in the main trial obtained the sub-study information. However, with the use of a standardized interview matrix, the risk of interviewer bias should be minor. Even though we were able to reach most patients, the patients who did not respond to a telephone call or letter reminder may represent patients less positive to study enrolment, which may signify selection bias. This reflects a common phenomenon in clinical research, where patients participating in trials are generally healthier and more positive to research [26]. We could not question the population who had declined participation (6.1%) in the original trial. Results from that group may have reflected a different experience of the IC process.

## Conclusion

Our results demonstrate that it is reasonable to ask patients for verbal IC in the acute phase of an AMI. The vast majority of patients felt positively about being asked and understood that they had been included in a clinical trial. Most patients disapproved of receiving information about the enrolment after randomization and treatment. Our results also indicate patient satisfaction with a routine of witnessed oral IC in the acute phase followed by written IC in the STEMI group.

## Data Availability

The datasets used and analysed during the current sub-study are available from the first author on reasonable request.

## References

[1] Cook DJ, Blythe D, Rischbieth A (2008). Enrollment of intensive care unit patients into clinical studies: a trinational survey of researchers’ experiences, beliefs, and practices. Crit Care Med.

[2] World Medical Association (2013). World Medical Association Declaration of Helsinki: ethical principles for medical research involving human subjects. JAMA.

[3] Berger BJ (2014). Minimum risk and HEAT-PPCI: innovative ideas for informed consent in emergency medical research. Ann Emerg Med.

[4] Dickert NW, Fehr AE, Llanos A, Scicluna VM, Samady H (2015). Patients’ views of consent for research enrollment during acute myocardial infarction. Acute Card Care.

[5] Zughaft D, Harnek J (2014). A review of the role of nurses and technicians in ST-elevation myocardial infarction (STEMI). EuroIntervention.

[6] Smith HL (1974). Myocardial infarction—case studies of ethics in the consent situation. Soc Sci Med.

[7] Foex BA (2004). Is informed consent possible in acute myocardial infarction?. Heart.

[8] Agard A, Herlitz J, Hermeren G (2004). Obtaining informed consent from patients in the early phase of acute myocardial infarction: physicians’ experiences and attitudes. Heart.

[9] Gammelgaard A (2004). Informed consent in acute myocardial infarction research. J Med Philos.

[10] Agard A, Hermeren G, Herlitz J (2001). Patients’ experiences of intervention trials on the treatment of myocardial infarction: is it time to adjust the informed consent procedure to the patient's capacity?. Heart.

[11] Gammelgaard A, Mortensen OS, Rossel P, DANAMI-2 Investigators (2004). Patients’ perceptions of informed consent in acute myocardial infarction research: a questionnaire based survey of the consent process in the DANAMI-2 trial. Heart.

[12] MacKay CR, Torguson R, Waksman R (2015). Delayed consent: will there be a shift in approach for US primary percutaneous coronary intervention trials?. Lancet.

[13] Shaw D (2014). HEAT-PPCI sheds light on consent in pragmatic trials. Lancet.

[14] Dickert NW, Hendershot KA, Speight CD, Fehr AE (2017). Patients’ views of consent in clinical trials for acute myocardial infarction: impact of trial design. J Med Ethics.

[15] Johnson LR, Siddaiah R (2015). Use of deferred consent for enrolment in trials is fraught with problems. BMJ.

[16] Shahzad Adeel, Kemp Ian, Mars Christine, Wilson Keith, Roome Claire, Cooper Rob, Andron Mohammed, Appleby Clare, Fisher Mike, Khand Aleem, Kunadian Babu, Mills Joseph D, Morris John L, Morrison William L, Munir Shahzad, Palmer Nick D, Perry Raphael A, Ramsdale David R, Velavan Periaswamy, Stables Rod H (2014). Unfractionated heparin versus bivalirudin in primary percutaneous coronary intervention (HEAT-PPCI): an open-label, single centre, randomised controlled trial. The Lancet.

[17] Erlinge D, Omerovic E, Frobert O (2017). Bivalirudin versus heparin monotherapy in myocardial infarction. N Engl J Med.

[18] Jernberg T, Attebring MF, Hambraeus K (2010). The Swedish Web-system for Enhancement and Development of Evidence-based care in Heart disease Evaluated According to Recommended Therapies (SWEDEHEART). Heart.

[19] Erlinge D, Koul S, Eriksson P (2016). Bivalirudin versus heparin in non-ST and ST-segment elevation myocardial infarction—a registry-based randomized clinical trial in the SWEDEHEART registry (the VALIDATE-SWEDEHEART trial). Am Heart J.

[20] Williams BF, French JK, White HD, HERO-2 investigators (2003). Informed consent during the clinical emergency of acute myocardial infarction (HERO-2 consent sub-study): a prospective observational study. Lancet.

[21] Andersen HR, Nielsen TT, Rasmussen K (2003). A comparison of coronary angioplasty with fibrinolytic therapy in acute myocardial infarction. N Engl J Med.

[22] Williams BF, French JK, White HD (1997). Is our method of obtaining consent appropriate for randomised controlled trials in acute myocardial infarction?. N Z Med J.

[23] Klein L, Moore J, Biros M (2018). A 20-year review: the use of exception from informed consent and waiver of informed consent in emergency research. Acad Emerg Med.

[24] Regulation (EU) No 536/2014 of the European Parliament and of the Council of 16 April 2014 on clinical trials on medicinal products for human use, and repealing Directive 2001/20/EC. https://eur-lex.europa.eu/eli/reg/2014/536/oj. Accessed 15 Dec 2019.

[25] Dal-Ré R, Avendaño-Solà C, Bloechl-Daum B, de Boer A, Eriksson S, Fuhr U (2019). Low risk pragmatic trials do not always require participants’ informed consent. BMJ.

[26] Kahan BC, Rehal S, Cro S (2015). Risk of selection bias in randomised trials. Trials.

